# Reliability analysis of the objective structured clinical examination using generalizability theory

**DOI:** 10.3402/meo.v21.31650

**Published:** 2016-08-18

**Authors:** Juan Andrés Trejo-Mejía, Melchor Sánchez-Mendiola, Ignacio Méndez-Ramírez, Adrián Martínez-González

**Affiliations:** 1 Secretariat of Medical Education, UNAM Faculty of Medicine, México City, México; 2 Institute of Applied Mathematics and Systems, UNAM, México City, México

**Keywords:** OSCE, reliability, generalizability theory, clinical competence, Mexico

## Abstract

**Background:**

The objective structured clinical examination (OSCE) is a widely used method for assessing clinical competence in health sciences education. Studies using this method have shown evidence of validity and reliability. There are no published studies of OSCE reliability measurement with generalizability theory (G-theory) in Latin America. The aims of this study were to assess the reliability of an OSCE in medical students using G-theory and explore its usefulness for quality improvement.

**Methods:**

An observational cross-sectional study was conducted at National Autonomous University of Mexico (UNAM) Faculty of Medicine in Mexico City. A total of 278 fifth-year medical students were assessed with an 18-station OSCE in a summative end-of-career final examination. There were four exam versions. G-theory with a crossover random effects design was used to identify the main sources of variance. Examiners, standardized patients, and cases were considered as a single facet of analysis.

**Results:**

The exam was applied to 278 medical students. The OSCE had a generalizability coefficient of 0.93. The major components of variance were stations, students, and residual error. The sites and the versions of the tests had minimum variance.

**Conclusions:**

Our study achieved a G coefficient similar to that found in other reports, which is acceptable for summative tests. G-theory allows the estimation of the magnitude of multiple sources of error and helps decision makers to determine the number of stations, test versions, and examiners needed to obtain reliable measurements.

The objective structured clinical examination (OSCE) is a widely used method for assessing clinical competence in health sciences education; it is considered the gold standard for this purpose, and there is abundant published evidence of validity and reliability (1). In 1975, Harden published the original version of the OSCE, using direct observation of students interacting with patients in multiple stations, to assess their clinical skills using checklists (2); it addressed some disadvantages of the traditional long-case examination, which has a low reliability (3).

The use of multiple stations in the OSCE is justified because the performance of a student in a single case is not a good predictor of performance in a different clinical situation, a phenomenon known as case specificity (4); thus, a large sample of clinical situations and longer testing time are required to achieve adequate reliability (5–7).

An important aspect of OSCE analysis and quality control is the measurement of reliability. The estimation of reliability in a test is a source of internal structure validity (8). ‘Reliability’ refers to the reproducibility of assessment data or scores, over time or occasions. It quantifies the consistency of the results and how reproducible they are when the test is applied in similar situations (5). The measurement of consistency and inconsistency in examinee performance constitutes the essence of reliability analysis (9). Measurement error always occurs, and the degree to which an individual score can vary from one test to another is known as the reliability coefficient (5, 9). It can be calculated using multiple approaches, such as Cronbach's alpha and generalizability theory (G-theory).

Cronbach's alpha estimates only one facet as a source of measurement error, that is, the scores on the stations; this imposes limitations on the estimation of the reliability of the OSCE. A facet represents a dimension or source of variation over which generalization is desired (9, 10). Since classical test theory has some limitations, Cronbach developed generalizability theory, which enables the calculation of multiple sources of error with a single measurement (11). It was initially used in behavioral sciences, education, and psychology, and later in medical education for assessment methods with many sources of variation, like the OSCE (7, 11). In the OSCE, a measurement error can be attributed to the following: 1) stations, 2) standardized patients, 3) examiners, 4) scenarios (if the OSCE is conducted at multiple sites), and 5) occasion effects (if the OSCE is conducted at different times).

Generalizability theory can be applied in formative and summative examinations, and its use is recommended to investigate the sources of error and the number of observations required for a given level of reliability (12, 13). Analysis of the sources of error in summative exams is useful as a quality control procedure to ensure reliable inferences from the results. G-theory assumes that the observed score of a person (the object of measurement) consists of a universal score (analogous to the true score in the classical test theory) and one or more sources of variation or facets (14).

G-theory begins by defining a finite universe consisting of observations on all possible levels of the factors that are of interest to the researcher. For example, if one is interested in estimating the contribution of examiners, versions, and stations for the measurement of communication skills, our universe is defined in terms of the number of levels of examiners, versions, and stations. Thus, the universal score is the average score of individuals across all levels of all factors in this specific finite universe (15).

G-theory uses the techniques of analysis of variance (ANOVA) for measurement in the behavioral sciences. It allows the estimation of the importance of factors such as examiners, students, and stations on the reliability of the OSCE and how different numbers of examiners, students, and stations change reliability (15). Two types of coefficients are used in G-theory: the G coefficient, which indicates the reliability level, and the dependability index (D), which is generally smaller than G. The D coefficient is used to design D studies to calculate the standard absolute error and its confidence intervals (14, 15).

The strengths of G-theory, which complement Cronbach's alpha, lie in its ability to identify which facets of the OSCE (stations, test versions, sites, or students) are the greatest source of measurement error. It also allows the decision maker to determine the number of examination occasions, the test formats, and the examiners needed to obtain reliable scores (14, 15). The scores obtained from an OSCE are vulnerable to potential errors because of seasonal variations, different versions, sites, and students. The monitoring these sources of variation functions as a quality control mechanism in order to ensure a valid interpretation of psychometric data and informed decision-making.

There are published reports of summative OSCE studies using G-theory, and the coefficients were between 0.51 and 0.78 (16–22). A large spread in G coefficients has been reported for formative OSCEs, ranging from 0.12 to 0.86 (7, 23, 24). All these studies were performed in developed countries, and there are no published replication studies from developing countries and Latin America. The goal of this study was to measure the reliability of a summative OSCE in a developing country medical school by using G-theory and to explore the usefulness of this approach for quality improvement.

## Method

### Subjects and setting

The available study population was 708 internship medical students from the National Autonomous University of Mexico (UNAM) Faculty of Medicine program. Most of the medical schools in Mexico have a 6-year program, of which the first 2 years include basic science courses, the next two and a half years comprise clinical courses, and the sixth year is an immersive full-time clinical experience in health care institutions called ‘medical internship’ (25). The internship rotation sites in our medical school are spread all over the country; therefore, we decided to use the available interns in the Mexico City area as sample for the study.

A 50% stratified random sample was selected to represent all internship hospitals and healthcare institution sites in Mexico City, which included 354 students. A total of 278 students (39.3% of the total population) took the summative end-of-career exam, which included the OSCE test used in our study. Their average age was 23.1 years: 72.7% were women and 27.3% were men. All students were familiar with the testing modality, as they had gone through a formative OSCE exam prior to the internship year.

### Design

This is a cross-sectional study with a summative OSCE assessment in the end-of-career graduating exam. The exam had four versions, each with 18 equivalent stations from six areas (pediatrics, obstetrics and gynecology, surgery, internal medicine, emergency medicine, and family medicine), for a total of 72 stations. The equivalent stations were designed from the same blueprint and explored similar clinical skills, although they were different cases. Efforts were made to explore similar problems and topics in the different versions of the test. The exam was applied in six clinical sites simultaneously during a 2-day period. The students were randomly distributed among the six sites.

We considered four facets or sources of variation: the students, the equivalent stations, the test versions, and the sites. The stations, the patients, and the examiners were considered a single facet of analysis because there was only one examiner in each station, and the patients did not evaluate the performance of the students. It was not possible to separate the effects of the station, the examiner, and the patient.

A generalizability study was carried out using a random effects design, in which the items used to determine reliability were the total scores of each student at each station (5). The dependent variable was the score obtained by students in the OSCE; the independent variables were the students, the stations, the sites, and the test versions.

### Case development

The components of clinical competence that were evaluated were 10 history-taking stations, three physical exam stations, one diagnosis and clinical management station, two radiographic interpretation stations, one laboratory studies interpretation, and one critical appraisal of a research paper station.

The assessment tools were developed and validated by a committee of experts on the six knowledge areas of the Medical Internship Program previously mentioned. The OSCE exam lasted for 2 h, with 6-min stations, and there were two rest stations. The raters evaluated the students with a station-specific checklist and marked the percentage of correct items for each station.

### Raters’ training

A total of 108 clinical teachers from the UNAM Faculty of Medicine participated in the examination process, 18 in each of the six clinical sites, that is, one examiner for each station. The students and the examiners did not know each other. All examiners were clinical faculty, with training and experience in the OSCE methodology.

The examiners reviewed the stations, the checklists, and the global rating scale of communication skills before the test. In 13 stations with patients, the examiner, apart from evaluating the checklist, assigned a score to the students’ communication skills using a global rating scale of 1 to 9 (1 to 3=unsatisfactory, 4 to 6=satisfactory, and 7 to 9=superior performance).

Each examiner used an op-scan sheet to score each student with the checklist and the global rating scale. All examiners attended the six sessions (three per day) and scored the four versions of the test.

### Standardized patients

A total of 124 patients participated in the examination; they were trained with a workshop and were evaluated with a formative OSCE 8 months before the exam. All the patients had participated in a minimum of five previous OSCE tests and were evaluated by the clinical raters. All standardized patients with an acceptable performance were included in the examination. Two standardized patients were excluded due to poor performance in the previous exams.

### Statistical analysis

**Fig. 1:**
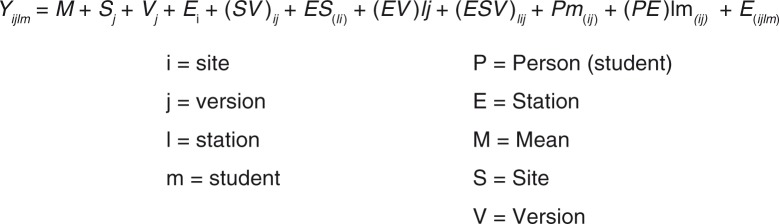
Model of statistical analysis to obtain variance component estimates to calculate the G coefficient.

Reliability tests with G-theory were carried out using the results of the ANOVA from the ‘model fit’ routine in JMP (SAS Software). The object of measurement, also called the facet of differentiation, was labeled ‘p’, for ‘person’ (Table 1). A random effects model was used to identify the main sources of variation. (Fig. 1) With this design, we obtained the estimates of the components of variance for each of the following facets: stations, sites, test versions, and all their interactions. We included the interaction ‘station×students’ nested within test versions and sites. Furthermore, we estimated the residual component of variance, which is the variability not explained by any of the facets.

**Table 1:** Notation system with G-theory

p:s^e^v×s
p=person (student or object of measurement)
s^e^=site
v=version
s=station
**:** means ‘nested within’
× means ‘crossed with’

In our OSCE study, the ‘p:s^e^v×s’ design means that students (*p*) are nested within the site (s^e^) and the test version (v) and that each student is crossed with each station (s). The test versions are crossed with the sites. The stations, in turn, are crossed with the sites and with the test versions (14, 15). We used JMP software (version 11) for the analysis of linear statistical models with both fixed and random effects. The formulas used for computing coefficients G and D are given in the Appendix.

### Ethical aspects and funding

The protocol for this study was approved by the Research and Ethics Committee of UNAM Faculty of Medicine and received support from the Program for Support of Projects for Innovation and Improvement of Teaching (PAPIME), UNAM, with project number PE207410. Participation in the study was voluntary. We preserved the confidentiality and anonymity of the students and present results in aggregate form.

## Results

The OSCE overall mean score was 63.2±5.7 (mean±SD). The distribution of the students among the exam versions was 97 in version1, 53 in version 2, 98 in version 3, and 30 in version 4.

The generalizability study revealed that the greatest source of variance (Table 2) was the residual error (51%). The stations were another source of variance (11.4%). No significant source of variance was found in the sites or in the test versions (0 and 0.2%, respectively).

**Table 2:** Variance component estimates obtained by analyzing the OSCE with G-theory (*n*=278)

Components	Variance component estimates	%
Station	42.157	11.4
Version	0.737	0.2
Version×station	38.968	10.6
Site	0	0
Site×station	46.631	12.7
Site×version	0.867	0.2
Site×station×version	34.692	9.4
Student [version, site]	17.652	4.8
Station×student [version, site]^a^	187.374	51
Total	367.024	100

Estimates based on equating mean squares to expected value. []=Nested,×=interaction.

a Residual error.

To determine the reliability of the test scores, we used the components of variance to calculate the G and D coefficients.

This design means that students (P) are nested within the site (S^e^) and the test version (V) and that each student is crossed with each station (S). The test versions are crossed with the sites. The stations, in turn, are crossed with the sites and with the test versions.

The G (generalizability) and D (Dependability) coefficients obtained from the analysis of the OSCE are shown in Table 3, with standard errors of measurement (SEM).

**Table 3:** G and D coefficients

	OSCE
G coefficient	0.93
SEM relative	1.16
D coefficient	0.83
SEM absolute	3.42

SEM=standard errors of measurement, OSCE=objective structured clinical examination.

## Discussion

This paper presents the results of an OSCE reliability study using G-theory. The OSCE can be improved by using G-theory because it provides information about the main indices of quality and validity evidence in the results of an assessment. The total variance of the OSCE was low; the reason might be that at the end of the internship year, and due to the similar educational process to which the students are subjected, they become more homogeneous (8, 14).

Regarding the estimates of the variance components in our OSCE, the students’ variance was 4.8%, which shows how much they differed in their performance.

The stations’ component of variance was 11.4%, which reflects the variance of the constant errors associated with levels of difficulty in the universe of the stations; the relative position of the students differed from one station to another.

The variation in the scores of the stations could be due to the OSCE content: history, physical examination, diagnosis and management, interpretation of radiographic and laboratory studies, and critical appraisal of a research paper (26).

The variance in the effect of the interaction between students and stations indicates that there are differences in the management of cases by the students. For example, a student might find it easy to manage some stations and may have difficulty to manage others. These results indicate that the difference in difficulty between stations differs from one student to another.

The interaction between versions and stations (10.6%) indicates that there are differences in the scores of stations according to the version. The interaction between the sites, versions, and stations (9.4%) is explained by the differences in the scores of stations according to the sites and versions.

Regarding the calculation of the G and D coefficients, an approach based on G-theory allows examining the implications of increasing or decreasing the size of the number of stations to assess its effect on the G coefficient. The D study, which is based on the findings of the G study, predicts what would happen if the number of stations is increased (15). In our study, an increase to 22 stations, with a duration of 2 h 24 min, would increase the G coefficient from 0.93 to 0.94. Versions and sites have a component of variance close to 0%; therefore, the increase in reliability would be marginal if they get changed.

All variances are considered to calculate the D coefficient (dependability), except those involving the student; the D coefficient was 0.83 (9, 15).

During the internship year, the students developed clinical competence; the lower variability of their components was reflected in the low values of the absolute and relative SEM of the OSCE (Table 3).

The G coefficient measures the proportion of the total variation produced by the variation in knowledge and skills of the students (18). A higher value of G implies that the other sources of variation are less important compared to the variation among students. Furthermore, the G coefficient value is considered to represent an acceptable reliability for a summative examination lasting 2 h (19). A meta-analysis found that the unweighted average of the generalizability coefficient was 0.49, as would be expected (27).

Lawson, Auewarakul, and Hatala conducted OSCEs and obtained a G coefficient from 0.62 to 0.68 (17, 19, 20). These values were lower than ours, which could strengthen our validity arguments.

Baig, Vallevand, Boulet, and Donnon also published OSCE studies and obtained a G coefficient from 0.51 to 0.78 (16, 18, 21, 22). These studies lasted longer but their G coefficient was equal to or lower than ours.

This study reports the experience of only one medical school in Mexico. We had only one examiner per station, which did not allow us to determine interexaminer reliability. It is recognized that G-theory is complex and requires expertise in its use (12).

There are several software programs that can be used to carry out generalizability studies, such as urGENOVA (15), SAS, G_String (13), and SPSS (14). G-theory involves a linear random effects model that is processed by JMP routines, which has the necessary tools to calculate estimates of the components of variance of studies with crossover and nested designs. In JMP, all you need is to put the scores (dependent variables) and all the individual facets, with interactions and nestings (independent variables), in the appropriate boxes, mark all of them as random, and run the program.

The reliability study using G-theory allowed us to identify several sources of variation that are involved in the OSCE, including the students’ variance and the residual used to calculate the G coefficient; it also allowed us to predict that increasing the number of stations would increase the reliability of the OSCE. The analysis of the stations will allow us to improve the quality of the OSCE.

The use of G-theory can solve some problems inherent to interexaminer reliability, such as overestimation of reliability, as pointed out by Fan (28). A large number of raters participated in our OSCE, and we made the reasonable assumption that any error due to differences between examiners was randomly distributed. We assume that the error of variance due to differences between examiners is small because they were all trained clinicians with standard performance in the OSCE.

Student characteristics such as gender and skill level were not considered in this study, even though they are potential sources of error; we recommend considering these variables in subsequent studies.

This is the first study to explore the use of OSCE in Latin America, using G-theory as a mathematical model for evaluating its reliability. One of the most important issues in modern research is the reproducibility of published studies, which has been debated exhaustively in many forums (29), and the situation is not improving due to a multitude of causes (e.g., publication bias, funding and emphasis on absolute originality, to name a few). The reproducibility problem has been studied in education research (30), where only 0.13% of education studies are replications. The reproducibility situation in medical education research has not been studied, but there is no reason to suspect it is any different from clinical, basic science, or general education research. The publication of results from different contexts, such as Latin American medical schools, can be relevant to the international medical community because it provides reproducibility evidence of the implementation and mathematical analysis of logistically complex and expensive assessment methods like the OSCE.

## Conclusions

The use of OSCE method in evaluating clinical competence has shown its usefulness. This assessment methodology has adequate reliability in our settings, and it could be of great importance to students, teachers, and medical schools using formative and summative OSCEs.

Equivalent versions of the examination, an appropriate planning, and rigorous implementation are factors that produced acceptable results. Our OSCE had good reliability as measured with G-theory.

Ensuring high quality of clinical competence assessment of students is the responsibility of all the relevant stakeholders, including the clinical teachers. The medical school authorities have an important responsibility in supporting and promoting the use of valid assessment methodologies.

## Appendix A

Formulas for Computing Coefficient G and Coefficient D

FORMULA FOR COEFFICIENT G

$$g=\frac{S_{p}^{2}}{S_{p}^{2}+\frac{\sigma_{pvs}^{2}}{n_{v}\times n_{s}}+\frac{\sigma_{pevs}^{2}}{n_{e}\times n_{v}\times n_{s}}}$$

where:

$S_{p}^{2}$=*student variance*

*n*_*s*_=*number of sites*

*n*_*v*_=*number of versions*

*n*_*e*_=*number of stations*

*n*_*e*_×*n*_*s*_=*number of stations*×*number of sites*

*n*_*v*_×*n*_*s*_=*number of versions*×*number of sites*

*n*_*e*_×*n*_*v*_×*n*_*s*_=*number of stations*×*number of versions*×*number of sites*

FORMULA FOR COEFFICIENT D

$$\Phi =\frac{S_{p}^{2}}{S_{p}^{2}+\frac{\sigma_{s}^{2}}{n_{s}}+\frac{\sigma_{v}^{2}}{n_{v}}+\frac{\sigma_{sv}^{2}}{n_{s}\times n_{v}}+\frac{\sigma_{e}^{2}}{n_{e}}+\frac{\sigma_{ev}^{2}}{n_{e}\times n_{v}}+\frac{\sigma_{es}^{2}}{n_{e}\times n_{s}}+\frac{\sigma_{evs}^{2}}{n_{e}\times n_{v}\times n_{s}}+\frac{\sigma_{pvs}^{2}}{n_{v}\times n_{s}}+\frac{\sigma_{pevs}^{2}}{n_{e}\times n_{v}\times n_{s}}}$$

## References

[1] Van der Vleuten CPM, Swanson DB (1990). Assessment of clinical skills with standardized patients: state of the art. Teach Learn Med.

[2] Harden RM, Stevenson WM, Downie W, Wilson GM (1975). Assessment of clinical competence using an objective structured clinical examination (OSCE). Br Med J.

[3] Hubbard JP (1971). Measuring medical education.

[4] Elstein AS, Shulman LS, Sprafka SA (1978). Medical problem solving.

[5] Downing S (2004). Reliability: on the reproducibility of assessment data. Med Educ.

[6] Petrusa ER, Norman GR, van der Vleuten CPM, Newble DI (2002). Clinical performance assessments. International handbook for research in medical education.

[7] Newble DI, Swanson DB (1988). Psychometric characteristics of the objective structured clinical examination. Med Educ.

[8] Downing SM (2003). Validity: on the meaningful interpretation of assessment data. Med Educ.

[9] Brennan RL (2010). Generalizability theory.

[10] Van der Vleuten C (2000). Validity of final examinations in undergraduate medical training. Br Med J.

[11] Cronbach L, Gleser G, Nanda H, Rajaratnam N (1972). The dependability of behavioral measurements: theory of generalizability for scores and profiles.

[12] Streiner DL, Norman G, Streiner DL, Norman G (2003). Reliability. Health measurement scales: a practical guide to their development and use.

[13] Cronbach L, Shavelson RJ (2004). My current thoughts on coefficient alpha and successor procedures. Educ Psychol Meas.

[14] Bloch R, Norman G (2012). Generalizability theory for the perplexed: a practical introduction and guide: AMEE Guide No. 68. Med Teach.

[15] Shavelson J, Webb N (1991). MMSS generalizability theory. A primer.

[16] Baig L, Violato C (2012). Temporal stability of objective structured clinical exams: a longitudinal study employing item response theory. BMC Med Educ.

[17] Lawson DM (2006). Applying generalizability theory to high-stakes objective structured clinical examinations in a naturalistic environment. J Manipulative Physiol Ther.

[18] Vallevand A, Violato C (2012). A predictive and construct validity study of a high-stakes objective clinical examination for assessing the clinical competence of international medical graduates. Teach Learn Med.

[19] Auewarakul C, Downing SM, Jaturatamrong U, Praditsuwan R (2005). Sources of validity evidence for an internal medicine student evaluation system: an evaluative study of assessment method. Med Educ.

[20] Hatala R, Marr S, Cuncic C, Bacchus CM (2011). Modification of an OSCE format to enhance patient continuity in a high-stakes assessment of clinical performance. BMC Med Educ.

[21] Boulet J, McKinley D, Whelan G, Hambleton R (2003). Quality assurance methods for performance-based assessments. Adv Health Sci Educ.

[22] Donnon T, Paolucci E (2008). A generalizability study of the medical judgment vignettes interview to assess students’ noncognitive attributes for medical school. BMC Med Educ.

[23] Hull AL, Hodder S, Berger B, Ginsberg D, Lindheim N, Quan J (1995). Validity of three clinical performance assessments of internal medicine clerks. Acad Med.

[24] Wilkinson TJ, Newble DI, Wilson PD, Cater JM, Helms RM (2000). Development of a three-centre simultaneous objective structured clinical examination. Med Educ.

[25] Sánchez M, Durante I, Morales S, Lozano R, Martínez A, Graue E (2011). Plan de Estudios 2010 de la Facultad de Medicina de la Universidad Nacional Autónoma de México. Gac Med Mex.

[26] Trejo J, Martínez A, Méndez I, Morales S, Ruíz L, Sánchez M (2014). Evaluación de la Competencia Clínica con el Examen Clínico Objetivo Estructurado (ECOE) en el Internado Médico de la UNAM. Gac Med Mex.

[27] Brannick MT (2011). A systematic review of the reliability of objective structured clinical examination scores. Med Educ.

[28] Fan X, Chen M (2000). Published studies of inter-rater reliability often overestimate reliability: computing the correct coefficient. Educ Psychol Meas.

[29] Open Science Collaboration (2015). Estimating the reproducibility of psychological science. Science.

[30] Makel MC (2014). Facts are more important than novelty. Replication in the education sciences. Educ Res.

